# Fees

**Published:** 1870-12

**Authors:** D. R. Jennings

**Affiliations:** Ravena, O.


### FEES.

BY D. R. JENNINGS, RAVENA, O.

Read before the Ohio State Dental Society.

The subject of "Fees for Dental Services," to which I invite attention, is one in which the whole profession is deeply interested, and yet has not received that degree of attention which its importance seems to demand.

I use the term fees, as it signifies something more than the amount of money received for operations; it means appreciation of skill and a reward for services. I do not believe that compensation for operations comes under the head of market prices; it is a reward for knowledge and skill, instead of a price for muscle; otherwise the coal heaver should command as high a price as the physician who has labored and studied to acquire a knowledge of the human system necessary to apply the proper remedies for the cure of disease.

The practice of underbidding can not be too strongly discountenanced. The man who underbids for the sake of getting practice away from another, often seriously injures himself, for the public soon learn that his operations correspond with the prices charged. We all know that the cheapest operations are the dearest in the end. The public value our services as we place a value upon them, as we judge ourselves the public will be sure to judge us. "The best is always the cheapest," holds good in dentistry as in everything else.

It is the duty of every Dentist to seek to elevate Dentistry and stand up for its reputation.

We must impress upon patients the fact that we do not charge for the amount of material we use, any more than the physician charges for the medicine he prescribes; but rather for the knowledge and skill required to complete the operation perfectly. Professional services admit of no positive regulation as to establishing a uniform rate of fees among practitioners. The only true rule to be governed by is qualification, skill and ability.

There will always be some to complain of the amount of their bills. We must expect that. But I tell such I would much rather have them find fault with my bill than with the operations.

I would like to say a few words in regard to fees for advice when no other service is rendered. It is an old and true saying that "an ounce of preventive is worth a pound of cure," and yet the larger number of Dentists make no charge for advice.

I think it should become a regulation among Dentists to charge for extracting when the patient intends to have a set of artificial teeth. It is often a tedious operation to the Dentist, requiring both patience and skill.

Why not charge for the surgical as well as the mechanical services rendered? The fault lies entirely with the Dentist, and it has always appeared wrong and ought to be abolished.

Another matter that lowers our profession in the eyes of many is that some operators in cases where they have filled several teeth for a patient, make no charge for cleaning or removing the tartar from the teeth, but "throw that in." Now this operation is oftentimes of as much or more benefit to the teeth than the fillings, and executed skillfully and judiciously requires considerable time and patience. I can see no reason why our services should be rendered gratuitously. From observations during a practice of fifteen years, I am of the opinion that as a general thing our patients are better satisfied and have a higher opinion of us when we charge them for every operation and do not endeavor to compliment them with gratuities.

The prevalence of cheap dentistry, low fees, and the small prospect of emoluments, induce many of our young men of brilliant talents, whom we should be glad to have with us, to enter other professions instead of the profession of dentistry. But while I am an advocate of liberal remuneration, I am also opposed to the extortion of exorbitant fees.

In conclusion I suggest three things that in my opinion prevent public confidence in dentistry:

*First.* The great number of poor and unsuccessful operations.

*Second.* The habit some Dentists have of finding fault with all operations not performed by themselves.

*Third.* The great irregularity in the fees charged by the profession in all parts of the country.

Taking these points into consideration, I wonder that the people are as liberal in their views of us as they are.

